# Design of User-Customized Negative Emotion Classifier Based on Feature Selection Using Physiological Signal Sensors

**DOI:** 10.3390/s18124253

**Published:** 2018-12-03

**Authors:** JeeEun Lee, Sun K. Yoo

**Affiliations:** 1 Graduate Program of Biomedical Engineering, Yonsei University, Seoul 03722, Korea; jeunlee@yuhs.ac; 2 Department of Medical Engineering, Yonsei University College of Medicine, Seoul 03722, Korea

**Keywords:** emotion, Kullback-Leibler divergence, physiological signal

## Abstract

First, the Likert scale and self-assessment manikin are used to provide emotion analogies, but they have limits for reflecting subjective factors. To solve this problem, we use physiological signals that show objective responses from cognitive status. The physiological signals used are electrocardiogram, skin temperature, and electrodermal activity (EDA). Second, the degree of emotion felt, and the related physiological signals, vary according to the individual. KLD calculates the difference in probability distribution shape patterns between two classes. Therefore, it is possible to analyze the relationship between physiological signals and emotion. As the result, features from EDA are important for distinguishing negative emotion in all subjects. In addition, the proposed feature selection algorithm showed an average accuracy of 92.5% and made it possible to improve the accuracy of negative emotion recognition.

## 1. Introduction

Recently, the number of people suffering from anxiety disorder has been increasing due to stress and irregular lifestyles. This negative emotion has a negative effect on human quality of life [1,2]. To apply emotion in various fields, it is necessary to know the exact definition of emotion. Emotion can be approached using basic or dimensional emotion theory. Basic emotion theory means there are universal basic emotions, regardless of culture, and that each emotion has unique characteristics. Among the basic emotions are interest, joy, surprise, sadness, fear, shyness, guilt, anger, disgust, and contempt. In contrast, dimensional emotion theory means that an individual emotion makes up a combination of a few dimensions rather than being a unique feature. It is expressed in valence-arousal space [3].

Among the various methods used to create an analogy of emotion are the Likert scale, self-assessment manikin (SAM), and text analysis. However, these methods reflect subjective reports by respondents. To overcome these limitations, we can use physiological signals that represent objective responses during the cognitive process. Therefore, physiological signals can be used to classify emotion [4,5]. Physiological signals are indicators that show responses of the central and autonomic nervous systems, and include electroencephalogram (EEG), electrocardiogram (ECG), skin temperature (SKT), and electrodermal activity (EDA).

Table 1 shows related studies about emotion classification using physiological signals. Almost all these classify arousal and valence based on dimensional emotion, because they are easy to score using a self-assessment manikin (SAM). SAM is a good way to evaluate mood, but is not suitable for dividing sections. Therefore, we use basic emotions such as No. 2, 3, and 8 (see Table 1) based on basic emotion theory. EEG was used to classify emotion status, as shown in Table 1. In particular, there are lots of studies about emotional response and frontal EEG asymmetry [6]. Therefore, EEG takes into account the most recent trends in mental state evaluations [7]. Additionally, electromyography (EMG) and electro-oculogram (EOG) signals were measured. These are suitable for the objective classification of emotion, especially fear, because they indicate facial expression. However, this would interfere with watching the videos. Specifically, EMG and EOG electrodes have to be attached to the face (near the eyes); so subjects could feel uncomfortable watching videos.

A physiological signal is difficult to interpret, because it is measured from fine current that is sensitive to interference from the external environment in the forms of crosstalk, measuring instruments, and movement artifacts [18]. Therefore, the experimental environment utilized is important, and signal processing and pre-processing are required to remove noise from the signals [19,20]. Also, when emotion is induced through video stimulation, the emotion is not continuously maintained because of variation in environmental factors such as the type of video, contents, and surroundings. If such problems occur, it becomes difficult for an emotion classifier to learn. Therefore, it is necessary to eliminate these factors by assuming they are outliers before classifier learning is conducted. In addition, when various features are extracted, the suitable and unsuitable features presented to a classifier are mixed. Moreover, the degree of emotion felt and the related physiological signals, vary according to the individual. It is difficult to generalize an emotion classification and there are limitations for improving classification accuracy using the same features. Therefore, selection of the feature to be used as input is important for classifying emotional status.

To solve this problem, there are feature selection algorithms such as the genetic algorithm, wrapper method, and restricted Boltzmann machine (RBM). The computational complexity of genetic algorithms is high, and they are difficult to apply to individuals [21]. The wrapper method, sequential backward selection (SBS) and sequential forward selection (SFS) are algorithms that delete or add features one by one. It is highly accurate, but there is a risk of over-fitting, and it is time-consuming [22,23]. To overcome these problems, a lot of techniques are used, such as information theory, resampling methods, cross-validation, etc. [24]. Also, principle component analysis (PCA), independent component analysis (ICA), swarm search and tabu search are used to avoid the curse of dimensionality [25]. Recently, deep learning methods such as RBM, auto-encoder, and deep belief networks (DBNs) have been used. These methods show excellent performance for classifying emotion, and solve the feature selection problem using hidden layers [26,27]. However, because the layers amount to a black-box, it is difficult to analyze the correlation between physiological signals and emotion. In this study, Kullback-Leibler Divergence (KLD) was used to solve these problems. KLD calculates the similarity of two different probability distributions [28]. It is used in cross-entropy functions to reduce training error by minimizing the negative log likelihood. In this case, KLD serves to measure how similar the probability distributions of the training output and target are [29].

On the other hand, for this study, we selected features with maximized KLD for negative emotion status. If KLD is large, a selected feature is significant for emotion classification, and it improves classifier performance. In addition, because KLD uses the shape pattern of probability distribution, it has better performance than when using the Gaussian distribution estimation method, if it is applied to probability distribution having complex characteristics using variational inference [30].

Therefore, the aim of this work was to design a user-customized negative emotion classifier based on KLD. We used physiological signals to reflect objective information about induced emotion. Moreover, Mahalanobis distance was used to exclude movement signals and the parts that did not indicate induced emotion. The features were selected according to KLD and information gain from the physiological signals acquired. The features selected show individual differences. Finally, the selected features were used as input to a negative emotion classifier in a neural network.

## 2. Experiments and Methods

### 2.1. Experimental Configuration and Data Acquisition

This experiment was conducted to induce emotion through visual stimulation and to acquire physiological signals according to emotion. Two types of visual stimulus were used. We used the horror movie “Saw 3” to induce negative emotion. “Two Faces of Humans” was used to induce basic emotion in the subjects. We used 60 min from the beginning of a movie to match the time of both videos equally. “Two Faces of Humans” is a psychology documentary related to human behavior in Korean society. This is most like a neutral stimulus because it is like the environment to which most Koreans are exposed in everyday life.

Written informed consent was obtained before the experiment. The subjects who participated in the experiment were fifteen men in their twenties (27 ± 2) who did not have mental or physical illnesses. The physiological signals of the subjects were sampled at 1 kHz using a BIOPAC MP 150TM instrument with ECG-100C, SKT-100C, and EDA-100C. The physiological signals measured were ECG, skin temperature, and electrodermal activity. Sensors were attached to the left seventh rib (+), under the right clavicle (−), and to the back of the neck (ground) for ECG measurement. The SKT was measured under the right arm and EDA was measured with a sensor attached to the middle and ring fingers of the right hand [31]. Before starting the experiment, subjects were instructed not to move during the experiment to minimize sensor noise from motion [32].

**Figure 1:**
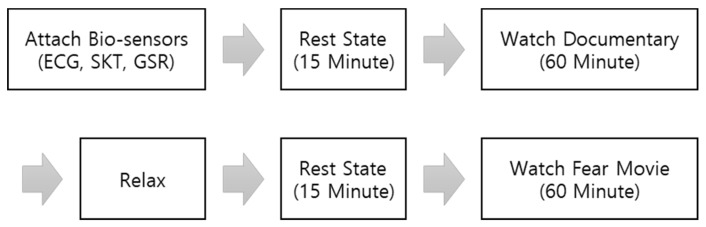
Experimental Protocol for Data Acquisition.

After attaching the sensors, two experiments were conducted according to the kind of visual stimulation. In the first experiment, the subjects were kept in a stable state for 15 min; then the documentary movie was shown for 60 min. After the first experiment, subjects took a period of rest adequate to cancel out the order effect; then the second experiment was started. After maintaining a stable condition for 15 min with a black screen, the subjects continued by watching a horror movie for 60 min. Because the documentary movie was closest to basic emotion, it was used first to minimize the order effect. Also, after watching each video, participants answered short questionnaires to check whether expected emotions is induced or not. The experimental protocol is shown in Figure 1.

### 2.2. Feature Extraction

The features from the measured physiological signal were extracted using a sliding window. The sliding window was fixed to five minutes and the overlap of windows was 30 s. The guideline for short-term heart rate variability (HRV) analysis is based on five minutes measurements by the Task Force of the European Society of Cardiology and the North American Society of pacing and Electrophysiology [33,34].

An ECG is a physiological signal that reflects the activity of the sympathetic and parasympathetic nerves of the autonomic nervous system. It is used to evaluate quantitatively the degree of activity of the autonomic nervous system. It was calculated by extracting the R peak using a QRS detection algorithm to extract features from the ECG. The features extracted from the time domain of the heart rate variability were the Mean HRV, the Standard Deviation of *NN* Intervals (*SDNN*), the mean value of the squared sum of heartbeat variances (*RMSSD*; the Square Root of the Mean Squared Difference Of Successive NNs), the number of times the heartbeat variability differed by more than 50 ms (*NN*50; the Number of Pairs of Successive NNs by Differential More Than 50 ms), and the ratio (*pNN*50; the Proportion Derived by Dividing *NN*50 by the NN Intervals). The latter was expressed using Equations (1)–(5). In the formula, “RR” means the length of heartbeat intervals, “*N*” means the number of heartbeat intervals, and “count” means number of heartbeats that occurred when the conditions within the parentheses were satisfied [15,35].

(1) $$MeanHRV=\sqrt{\frac{1}{N}\sum_{i=1}^{N}{\left(RR_{i}\right)}^{2}}$$

(2) $$SDNN=\sqrt{\frac{1}{N-1}\sum_{i=1}^{N}{\left(RR_{i}-MeanHRV\right)}^{2}}$$

(3) $$RMSSD=\sqrt{\frac{1}{N-1}\sum_{i=1}^{N}{\left(RR_{i}-RR_{i-1}\right)}^{2}}$$

(4) $$NN50=Count(RR_{i}>50ms)$$

(5) $$pNN50=\frac{NN50}{N}\times 100\%$$

The features extracted from the frequency domain are robust in fear, but weaker in happiness [36]. The extracted features were the ratio of low frequency to high frequency power (LF/HF), total spectral power (TP) in the range 0.003–0.4 Hz, normalized high-frequency power (nHF) in the range 0.15–0.4 Hz, and normalized low-frequency power (nLF) in the range 0.04–0.15 Hz [35].

The SKT is another physiological signal influenced by the autonomic nervous system. The rate of response to the stimulus is fast. The pre-processing method for collecting the SKT signal was used: the signal was down-sampled at 50 Hz and then passed through a low-pass filter to remove noise [37]. The SKT features were calculated from raw signals into each of five minute long windows. The features extracted from the SKT were Mean SKT (Mean Skin Temperature) and Standard Deviation of SKT (SD SKT).

The EDA index is affected by the sympathetic nervous system. The EDA requires signal processing because of its high dynamic characteristics and high sensitivity to noise. Thus, the acquired EDA signal was down-sampled at 50 Hz. The high-frequency components were removed. The EDA includes a tonic component representing skin conductance level (SCL) and a phasic component representing skin conductance response (SCR) [38,39]. The tonic and phasic components were separated using discrete wavelet transforms. The mother wavelet uses a third-order Daubechies wavelet (db3) that best represents the original EDA signal. The frequency bandwidth corresponding to 32 Hz is approximated through the discrete wavelet decomposition process. The highest approximation level factor (Decomposition level: A10, Frequency bandwidth: 0–0.015625 Hz) representing the low-frequency component is the tonic component, which is the essence of electrodermal activity. The phasic component was calculated by subtracting the tonic component from the original EDA. The extracted features are tonic and phasic components of the zero crossing (ZC EDAP) and standard deviation (SD EDAP) of the phasic components; and the mean, standard deviation (SD EDAT), and amplitude (Amp EDAT) of the tonic components of the EDA [40].

The feature vector consisted of 16 features: nine within the ECG signal, two within the SKT signal, and five within the EDA signal. Assuming that emotion was not induced at the beginning of the visual stimulus, 100 windows were selected as representative feature vectors of each emotion at the end of the visual stimulus. Each window makes one feature vector. Therefore, 100 feature vectors were extracted from the basic emotion data and 100 feature vectors from the negative emotion data for each subject. Thus, a total of 200 feature vectors were used. The value of each extracted feature was normalized between “0” and “1”.

### 2.3. Feature Selection

Even though the same visual stimuli were given, the degree of emotion felt was different, and emotional induction might also not occur, depending on the subject. Also, in cases of signal noise from motion artifacts or non-induced emotion status, the feature vectors reflected the situation. When emotion was not induced, the data were outliers. The Mahalanobis distance was calculated to remove outliers.

The KLD and information gain were calculated for each feature value to extract the upper values that affect the discrimination of negative emotion among the 16 extracted features. KLD is a method of calculating the distance between probability distributions of each class. During the sampling process, it computes entropy differences when using *q*(*x*), which is the approximate probability distribution of *p*(*x*) instead of the actual probability distribution of *p*(*x*). Equation (6) represents the KLD, as $D_{KL}$, *x* is data of a set *S*, the term p represents the probability distribution, and q represents the approximate probability distribution of *p*(*x*). Equation (6) is a method of calculating the Kullback-Leibler Divergence, defined as the cross entropy of *p*(*x*) and *q*(*x*) subtracting the entropy of *p*(*x*). In this study, it was used to select feature values for classification [28,41].

(6) $$D_{KL}(p||q)=E\left[logp\left(x\right)-logq\left(x\right)\right]=\sum_{x\in S}p\left(x\right)\cdot log\frac{p\left(x\right)}{q\left(x\right)}$$

In addition, the information gain was calculated from Equations (7) and (8) for each feature. Equation (7) represents the entropy of the set S. Equation (8) represents the information gain by calculating the difference of entropy when the lower node A is selected from the entropy of the upper node, and T represents the number of lower nodes. Here, t represents each of the lower nodes [42]. The information gain values calculated from Equation (8) are sorted in descending order of importance and indicate the feature selected as input up to the convergence point. Larger KLD indicates that the feature is more important. The features are sorted in descending order of importance. The information gain is calculated by Equation (8) by iteration, adding the features one-by-one. When the information gain is converged, the feature is selected as input up to the convergence point. The convergence point was calculated from Equation (9). Equation (9) represents the maximization value of the information gain “max(IG(A, S))”, minimization value of the value of information gain “min(IG(A, S))”, and the differential value ‘diff’. The features were optimized with argument maximization until the information gain converged. Thus, a User-Customized automatic feature selector was designed using the feature-selection algorithm.

(7) $$H\left(S\right)=-\sum_{x\in S}p\left(x\right)\log_{2}p\left(x\right)$$

(8) $$IG\left(A,S\right)=H\left(S\right)-\sum_{t\in T}p\left(t\right)H\left(t\right)$$

(9) $$CP=\frac{1}{4}(\max\left(IG\left(A,S\right)\right)-\min\left(IG\left(A,S\right)\right)(if,\;diff\left(IG\left(A,S\right)\right)<\frac{1}{2}\max\left(diff\left(IG\left(A,S\right)\right)\right))$$

### 2.4. Design of the Neural Network Classifier

In this study, neural networks were used to evaluate the performance of the model from the feature selected according to individual subjects. Because the number of data extracted from one individual is small, the validity of the model was verified using the leave-one-out cross-validation (LOOCV) method. The model was trained with (N − 1) of the total data samples (n), and the model was evaluated with the one remaining sample. LOOCV is a method for model validation in which the process is repeated n times. It is useful for small data sets because it reduces bias and prevents overly optimistic predictions [43,44].

A neural network (NN) can play a role in classifying input data, even for complicated input, provided it is given an adequate learning process. In this study, we used a multi-layer perceptron with one hidden layer, because more than two hidden layers causes a vanishing gradient problem. The neural networks were trained using a back-propagation algorithm to optimize the weight of various features. A NN should be designed for the highest performance by varying conditions such as the number of hidden layers, number of hidden nodes, and learning rate [45]. In this study, we designed the same NN except for the number of input layers, to provide the highest accuracy, on average, for all subjects. The final design was a NN with one hidden layer, a hidden node (input feature number − 1), learning rate of 0.01, repetition frequency of 2000, and sigmoid activation function.

## 3. Results

### 3.1. Outlier Removal Results of Each Feature

Through the survey, we checked that emotion was induced in all of the subjects. On average, 29 outliers were removed per subject. Table 2 shows the classification accuracy of each feature before and after outlier removal. 

The accuracy was improved for most features after outlier removal (on average), except for three features (LF/HF, nHF and nLF). In particular, the frequency domain features extracted from the electrocardiogram show that performance after removal of the outliers was worse. The standard deviations for the features extracted from the measured electrocardiogram made during each visual stimulus were small and within the same range. Moreover, the means for the features extracted from the measured electrocardiogram were the same. For this reason, outliers were not removed, and the probability distributions of the statuses overlapped. In addition, the features show a performance difference of less than 1% before and after outlier removal, so the difference was not significant. Regarding electrodermal activity, Table 2 shows that the accuracy improved for all features after removing the outliers. As shown in Table 2, the classification accuracy was higher for all features of electrodermal activity. It is shown that the removal of outlier data is a preprocessing factor that enhances algorithm performance.

### 3.2. Selected Features

**Figure 2:**
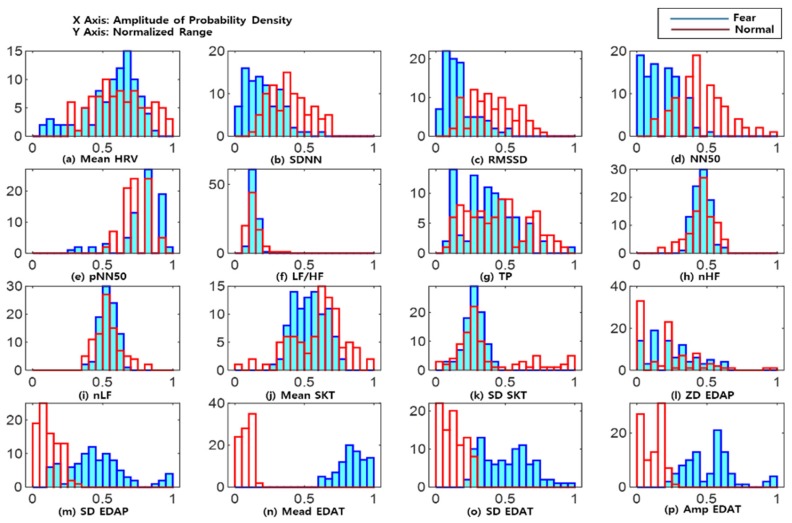
Histograms of the probability distribution of (**a**) Mean HRV, (**b**) SDNN, (**c**) RMSSD, (**d**) NN50, (**e**) pNN50, (**f**) LF/HF, (**g**) TP, (**h**) nHF, (**i**) nLF, (**j**) Mean SKT, (**k**) SD SKT, (**l**) ZD EDAP, (**m**) SD EDAP, (**n**) Mean EDAT, (**o**) SD EDAT, and (**p**) Amp EDAT.

Figure 2 shows histograms of the probability distribution of each feature value extracted from the basic and fear emotions of one subject. It shows the shape pattern of the probability distribution. In Figure 2, the blue bars represents the probability distribution of the fear emotion, the red bars represent the probability distribution of the basic emotion, the x-axis represents the range of the normalized characteristic values between “0” and “1”, and the y-axis represents the number of feature values belonging to the range. The KLD was calculated from the probability distribution of the extracted features, and the importance of the feature values was determined based on how far apart the maximum point of probability distributions between the two emotions were.

Table 3 shows features from the physiological signals selected through the KLD and information gain. The common feature was Mean EDAT, and this was comparable with other selected features, including SD EDAP, SD EDAT, and Amp EDAT. These features were mostly extracted from the EDA signals. As shown in the probability distribution of the features in Figure 2, the KLD was highest for electrodermal activity. In addition, from Table 3, it can be seen that the time domain features comparing frequency domain features extracted from the ECG according to subject, were more used as inputs. The features selected from the ECG, the skin temperature, and the EDA signals, were all used. The characteristics of each individual were slightly different, but all of the measured physiological signals changed when negative emotion was induced.

### 3.3. Comparison of Classification Accuracy According to Features

Equations (10)–(14) represent accuracy, sensitivity, specificity, positive predictive value (PPV), and negative predictive value (NPV), respectively. The true positive (TP) was classified as negative emotion when watching “Saw 3”. True negative (TN) was classified as neutral emotion when watching “Two Faces of Humans”. The false positive (FP) was classified as neutral emotion when watching “Saw 3”, and the false negative (FN) was classified as negative emotion when watching “Two Faces of Humans”.

(10) $$\mathrm{Accuracy}=\frac{TP+TN}{TP+FN+FP+TN}$$

(11) $$\mathrm{Sensitivity}=\frac{TP}{TP+FN}$$

(12) $$\mathrm{Specificity}=\frac{TN}{FP+TN}$$

(13) $$\mathrm{PPV}=\frac{TP}{TP+FP}$$

(14) $$\mathrm{NPV}=\frac{TN}{FN+TN}$$

Table 4 shows comparison of the accuracy before and after feature selection. In addition, one of the most important features selected from KLD is compared. Table 4 shows the accuracy, sensitivity, specificity, PPV, and NPV using all the feature vectors and using the selected feature vectors.

When all the features were used as inputs, they had an accuracy of 87.3%, on average. On the other hand, when only the features selected using the feature selection algorithm were used as inputs, the average accuracy was 92.5%. On average, use of a single feature had an accuracy of 82.6%. When the feature selection algorithm proposed in this study was used, the accuracy of negative emotion classification increased for all subjects, and the accuracy of the classifier was improved by about 5.2% compared with using all the features, on average. Using one feature had the lowest accuracy and showed a performance difference about 10% less than when using selected features.

In addition, we confirmed that the sensitivity and specificity increased after selecting features, and the reliability of the developed model also increased. PPV means that if the classifier detects fear (91.7%), the real probability of feeling negative emotion is 93.3%. When sensitivity and PPV, and specificity and NPV are high, the reliability is greater. In the case of using one feature, sensitivity had the highest value. This means that when negative emotion is induced, one of the most important features classifies negative emotion with the best accuracy. In contrast, sensitivity and PPV had the lowest values. This means that when basic emotion is induced, the classifier accuracy is decreased.

### 3.4. Comparison Performance of Neural Network and Other Classifiers

Table 5 shows the comparison of the accuracy according to classifiers. We used linear discriminant analysis (LDA) and quadratic discriminant analysis (QDA) to compare performance with the NN that we selected. The classifiers were trained with the selected features. Each class had the same covariance matrix in the LDA. In contrast, the QDA assumed that each class had a distinct covariance matrix [46]. Table 4 shows the accuracy, sensitivity, specificity, PPV, and NPV using each classifier.

When the NN was used, accuracy was 92.5%, on average. The accuracy of LDA and QDA was 83.5% and 85.6%, on average. When the NN proposed in this study was used, the accuracy of negative emotion classification was highest, and the accuracy of the classifier was improved by about 9% compared with using LDA, on average. Table 5 shows that when the classifier model is more complex, the performance is better.

In addition, we confirmed that the sensitivity, PPV, and NPV increased by using the NN. However, when we use LDA, specificity was at its highest. This means that LDA classified negative emotion better than other classifiers did, but its sensitivity was the lowest. This means its performance at classifying basic emotion was lower than with the other options.

## 4. Discussion

In this paper, we proposed a negative emotion classifier that combines a feature selection algorithm using Kullback-Leibler Divergence, with information gain and a neural network. These were used to process physiological signals acquired through emotion induction, after pre-processing by outlier removal using Mahalanobis distance.

It is difficult to quantify the degree of emotion because individual deviations are different for feeling emotion and because there is a large subjective factor depending on the stimulus. Therefore, in this study, rather than quantification of emotion, we tried to induce emotion using visual stimuli (documentary, horror movie) and then classify the negative emotions.

If visual stimulation is used, it is assumed that emotion is maintained according to the mood induced by the stimulus. However, it cannot be confirmed whether the mood induced by intentional stimulation is sustained in the subject. In addition, because physiological signals are sensitive to signal noise such as motion, there is a possibility that noise is mixed into the acquired data. Therefore, it is necessary to remove outliers before classifier design. Assuming that the feature vector has a normal distribution according to emotion, data that are far away from the normal distribution are removed and the well-derived emotion data are used to train the classifier. The classification performance is improved by eliminating the outliers, as shown Table 2. Outliers were also eliminated from random sections of the videos used as stimuli. This means that movement occurred randomly and that the emotion induced, differed by subject. Thus, the outlier elimination algorithm resolves issues caused by environmental variability such as state of emotion induction and movement noise.

The physiological signals were used as inputs to reflect objective factors. In the case of ECG, the comparison between the HRV indices obtained with different measurement times is prohibited, and indices obtained at the same measurement time must be compared [34]. Therefore, a window size for five minutes was used for further comparison with other studies. Results from earlier studies show that when negative emotion is induced, the heart rate, SCR, and SCL of EDA increase, and that SKT decreases [34,38]. As shown in Table 3, EDA played an important role in measuring negative emotion. Because EDA has the characteristic of being sensitive to the degree of stress [38], it is considered to have great influence on the detection of negative emotion, in comparison with other physiological signals. The ECG showed that different features were extracted according to subjects, but that these changed when negative emotion was induced. SKT was expected to be affected by temperature in the laboratory because it was sensitive to the ambient environment [39]. The gain of physiological signals is also different according to subject. For example, some subjects have stronger ECG signals, even when electrodes are attached in the same positions. This makes the relevance of features different, as shown in Table 3. RMSSD, SDNN, and Mean EDAT are estimated with large weighting to classify negative emotion, but the relevance could be changed by the environment or by the body structure of the subject. In the future, when discriminating negative emotion by inputting a physiological signal, EDA could be used for design of a high-performance classifier.

In this study, because physiological signals differ from person to person, a user-customized feature selection classifier was designed. Also, it cannot be confirmed whether the mood induced by intentional stimulation is sustained in the subject. In addition, because physiological signals are sensitive to signal noise such as motion, there is a possibility that noise is mixed into the acquired data. Therefore, it is necessary to remove outliers before classifier design. Surveys reflect subjective opinions, but they can be manipulated according to intentions. It is also difficult to instantaneously survey what someone feels while watching a movie. This could be a factor that hinders emotional induction. Therefore, the survey was used just to check whether the subject felt the emotion induced in the experiment, and the section of induction of emotion by the time was selected using the Mahalanobis distance. After removal of outliers, assuming that the feature vector has a normal distribution according to emotion, data that are far away from the normal distribution are removed and the well-derived emotion data are used to train the classifier. Also, features were selected for each subject using the higher values from calculation of KLD and information gain. KLD can be used as a feature selection algorithm because it can calculate the difference in probability distributions between two classes [26]. KLD shows differences in the probability distribution shape patterns. Using the KLD is better than assuming a Gaussian distribution for known data characteristics. The KLD is expressed using variational inference, so it is more accurate than a Gaussian approximation, even if two probability distributions have the same mean and standard deviation. The KLD has the advantage that it can directly check the validity of each feature by using it with information gain. Although an RBM does not have information on the extracted features, the feature selection method in this paper could make known the information about the extracted features [25]. Thus, it could analyze the relationship between physiological signals and emotions. It is also possible to simplify the system and generate a model in the future. In addition, because the weighting of the selected feature is adjusted again while learning the neural network, a better classifier is designed when all the features are used. SD EDAP, SD EDAT, and Amp EDAT, which are important features extracted from the EDA signal, have high accuracy in classifying negative emotion with high information gain. They are also useful after outlier removal with other features. The results show that it is important not only to design a classifier, but also to select the input feature vectors carefully to improve classifier performance. Using a variety of features for input can improve the performance of the classifier, but mixing the noise can result in complicated calculations as well as degradation of the classifier. Each individual (subject) has unique physiological signal characteristics. This means that selection of an appropriate feature plays an important role in improving performance of the classifier when physiological signals are used to derive features.

Finally, we used a neural network because it consists of summation of linear algebra and activation function; thus, it is easier to use than other machine learning methods such as support vector machine (SVM). The NN also has the best performance compared with the other classifiers tested (LDA and QDA). LDA and QDA are easier to calculate and more intuitive than other deep learning methods are. These results confirm that when the classifier model is more complex, the performance is better. Various classifiers are used to classify emotion including such as K-nearest neighbor (KNN), random forest (RF), convolutional neural networks (CNN), and long-short term memory (LSTM) [18]. The SVM is the most used classifier, as shown in Table 1. The highest accuracy is exhibited with a Bayesian network. The G-extreme Learning Machine has accuracy of 91.1%. The accuracy of the classifiers depends on many factors such as the number of classes, the physiological signal used, and the features used. Therefore, we should apply various machine learning methods to improve classification performance.

## 5. Conclusions

The proposed feature selection algorithm allowed to improve accuracy of negative emotion recognition with data from fifteen subjects and fixed video stimuli. Therefore, further studies are required to confirm whether the algorithm can be effectively applied after increasing the number of subjects. especially, to evaluate the feature selection algorithm, the paired *t*-test will be applied with sixteen more subjects according to the value of effective size from the result from fifteen subjects, power (0.9), significance level (*p* < 0.05). The classifier has the possibility of overfitting because it has been trained with individual data. Therefore, further research is needed to verify whether the selected features from the same individual are effective for prediction even when watching a new video stimulus to induce negative emotion. In addition, we could improve performance of the algorithm if it were optimized by automatically changing the fixed parameters in the process of designing the neural network. We used a NN because it has high accuracy with simple structure. However, complex models such as deep learning have high accuracy and recently; many deep learning methods have been developed. Therefore, we could apply the results from other recent studies to classify negative emotion and compare those results with the method we used. Moreover, applying the algorithm of feature selection and classification starting from three different initial feature-sets (only HRV features, only SKT features, and only EDA features), it is clear that a different feature extraction method (e.g., a model-based approach) and more features are needed for real environmental implementation.

## Figures and Tables

**Table 1:** Other studies about classification of emotion using physiological signals.

No.	Emotions	Signals	Classifiers	Accuracy [%]
1 [8]	Arousal, Valence	EEG	SVM	82.0
2 [9]	Amusement, Fear, Sadness, Joy, Anger, Disgust	EEG, ECG	Bayesian Network	98.1
3 [10]	Amusement, Grief, Anger, Fear, Baseline	OXY, GSR, ECG	RF	74.0
4 [11]	Arousal, Valence	EMG, RSP	SVM	74.0
5 [12]	Arousal, Valence	EEG	LSTM	72.1 for valance 74.1 for arousal
6 [13]	Arousal, Valence	EEG	KNN, RF	69.9 for valance 71.2 for arousal
7 [14]	Arousal, Valence	EEG, EMG, EOG, GSR, RSP, T, BVP	SVM	88.3 for valence 90.6 for arousal
8 [15]	Positive, Negative	ECG	SVM	73.1
9 [16]	Arousal, Valence	EEG	G extreme Learning Machine	91.1
10 [17]	Happy, Curious, Angry, Sad, Quiet	EEG	QDA	47.5

**Table 2:** Accuracy comparison result according to outlier reduction.

Feature	Before Outlier Reduction	After Outlier Reduction
Mean HRV [%]	50.37	51.16
SDNN [%]	52.77	54.48
RMSSD [%]	52.60	54.37
NN50 [%]	52.17	54.81
pNN50 [%]	50.57	51.86
LF/HF [%]	49.87	49.10
TP [%]	49.13	52.56
nHF [%]	50.60	49.88
nLF [%]	50.6	49.88
Mean SKT [%]	47.20	52.31
SD SKT [%]	49.13	50.67
ZC EDAP [%]	52.47	54.62
SD EDAP [%]	59.80	64.82
Mean EDAT [%]	70.87	74.90
SD EDAT [%]	61.57	65.77
Amp EDAT [%]	61.57	66.64

**Table 3:** Selected features from the proposed feature selection algorithm.

Subject No.	Selected Features
1	Mean EDAT, SD EDAP, SD SKT, Amp EDAT, SD EDAT, RMSSD, NN50
2	Mean EDAT, SD EDAP, SD SKT, Amp EDAT, SD EDAT, TP
3	RMSSD, SDNN, Amp EDAT, SD EDAP, Mean EDAT, SD SKT, SD EDAT, NN50
4	RMSSD, SDNN, SD EDAP, Mean EDAT, Amp EDAT, SD EDAT, SD SKT, LF/HF
5	SDNN, RMSSD, SD EDAP, Mean EDAT, SD SKT, Amp EDAT, SD EDAT, LF/HF
6	Mean EDAT, Amp EDAT, RMSSD, Amp EDAT, SDNN, SD EDAT, SD SKT, ZC EDAP, TP, NN50, pNN50, nHF, nLF, Mean HRV, Mean SKT, LF/HF
7	RMSSD, SDNN, Mean EDAT, SD EDAP, Mean SKT, SD SKT, SD EDAT, ZC EDAP, Amp EDAT, TP, NN50, nHF, nLF, LF/HF, Mean HRV, pNN50
8	RMSSD, SDNN, SD EDAP, Mean EDAT, Amp EDAT, SD EDAT, nLF
9	Mean EDAT, Amp EDAT, LF/HF, SD EDAT, nLF, nHF, TP, SD EDAP, RMSSD, NN50
10	SD EDAP, RMSSD, Mean EDAT, SDNN, Amp EDAT, SD EDAT, TP, LF/HF, SD SKT, nLF, nHF, NN50, ZC EDAP, pNN50, Mean HRV, Mean SKT
11	nHF, nLF, LF/HF, RMSSD, TP, SDNN, Mean EDAT, Mean SKT
12	nHF, nLF, Mean EDAT, TP, ZC EDAP
13	SD EDAT, SD EDAP, Mean EDAT, nHF, nLF, RMSSD, Mean HRV, LF/HF, pNN50
14	Mean EDAT, nHF, nLF, LF/HF, pNN50, Mean HRV
15	SDNN, RMSSD, Mean EDAT, SD EDAP, SD SKT, LF/HF, TP, Mean HRV

**Table 4:** Statistical analysis according to features.

Value	All Features [%]	Selected Features [%]	One Feature [%]
Accuracy	87.3	92.5	82.6
Sensitivity	86.3	91.7	92.5
Specificity	88.3	93.3	72.6
Positive Predictive Value	90.6	93.3	77.5
Negative Predictive Value	89.1	91.9	90.3

**Table 5:** Statistical analysis according to classifiers.

Value	NN [%]	LDA [%]	QDA [%]
Accuracy	92.5	81.2	85.6
Sensitivity	91.7	92.1	84.7
Specificity	93.3	70.1	86.5
Positive Predictive Value	93.3	76.1	86.6
Negative Predictive Value	91.9	89.5	85.3
